# Autoimmune and Autoinflammatory Connective Tissue Disorders Following COVID-19

**DOI:** 10.1001/jamanetworkopen.2023.36120

**Published:** 2023-10-06

**Authors:** Sung Ha Lim, Hyun Jeong Ju, Ju Hee Han, Ji Hae Lee, Won-Soo Lee, Jung Min Bae, Solam Lee

**Affiliations:** 1 Department of Dermatology, Yonsei University Wonju College of Medicine, Wonju, Republic of Korea; 2 Department of Dermatology, St Vincent’s Hospital, College of Medicine, The Catholic University of Korea, Suwon, Korea; 3 Department of Dermatology, Seoul St Mary’s Hospital College of Medicine, The Catholic University of Korea, Seoul, Korea; 4 Department of Preventive Medicine, Yonsei University Wonju College of Medicine, Wonju, Republic of Korea

## Abstract

**Importance:**

Multiple cases of autoimmune and autoinflammatory diseases after COVID-19 have been reported. However, their incidences and risks have rarely been quantified.

**Objective:**

To investigate the incidences and risks of autoimmune and autoinflammatory connective tissue disorders after COVID-19.

**Design, Setting, and Participants:**

This was a retrospective population-based study conducted between October 8, 2020, and December 31, 2021, that used nationwide data from the Korea Disease Control and Prevention Agency COVID-19 National Health Insurance Service cohort and included individuals who received a diagnosis of COVID-19 via polymerase chain reaction testing and a control group with no evidence of COVID-19 identified from National Health Insurance Service of Korea cohort. Data analysis was conducted from September 2022 to August 2023.

**Exposures:**

Receipt of diagnosis of COVID-19.

**Main Outcomes and Measures:**

The primary outcomes were the incidence and risk of autoimmune and autoinflammatory connective tissue disorders following COVID-19. A total of 32 covariates, including demographics, socioeconomic statuses, lifestyle factors, and comorbidity profiles, were balanced through inverse probability weighting. The incidences and risks of autoimmune and autoinflammatory connective tissue disorders were compared between the groups using multivariable Cox proportional hazard analyses.

**Results:**

A total of 354 527 individuals with COVID-19 (mean [SD] age, 52.24 [15.55] years; 179 041 women [50.50%]) and 6 134 940 controls (mean [SD] age, 52.05 [15.63] years; 3 074 573 women [50.12%]) were included. The risks of alopecia areata (adjusted hazard ratio [aHR], 1.12; 95% CI, 1.05-1.19), alopecia totalis (aHR, 1.74; 95% CI, 1.39-2.17), antineutrophil cytoplasmic antibody–associated vasculitis (aHR, 2.76; 95% CI, 1.64-4.65), Crohn disease (aHR, 1.68; 95% CI, 1.31-2.15), and sarcoidosis (aHR, 1.59; 95% CI, 1.00-2.52) were higher in the COVID-19 group. The risks of alopecia totalis, psoriasis, vitiligo, vasculitis, Crohn disease, ulcerative colitis, rheumatoid arthritis, adult-onset Still disease, Sjögren syndrome, ankylosing spondylitis, and sarcoidosis were associated with the severity of COVID-19.

**Conclusions and Relevance:**

In this retrospective cohort study, COVID-19 was associated with a substantial risk for autoimmune and autoinflammatory connective tissue disorders, indicating that long-term management of patients with COVID-19 should include evaluation for such disorders.

## Key Points

**Question:**

Is COVID-19 associated with an increased risk of autoimmune and autoinflammatory disorders?

**Findings:**

This cohort study including 354 527 individuals with COVID-19 and 6 134 940 controls identified a significant elevation in the risk of multiple incident autoimmune and autoinflammatory disorders subsequent to COVID-19. Notably, certain disease risks exhibited a positive association with the severity of COVID-19.

**Meaning:**

These findings suggest that autoimmune and autoinflammatory connective tissue disorders may manifest as post–COVID-19 sequelae, highlighting the potential long-term health ramifications associated with COVID-19; long-term management should include evaluating the development of such disorders in patients who had COVID-19.

## Teaser

This cohort study analyzes the risk of incident autoimmune and autoinflammatory connective tissue disorder following COVID-19.

## Introduction

COVID-19 is widespread, and its association with various other diseases have been reported.^1,2,3,4,5,6^ Possible associations of COVID-19 with autoimmune diseases also have been suggested,^7^ because SARS-CoV-2 appears to perturb self-tolerance and trigger autoimmune reactions via cross-reactivity that may lead to the development of autoimmune diseases.^6^ A growing body of literature^7,8,9^ has reported various disease cases—including alopecia areata, vitiligo, systemic lupus erythematosus (SLE), vasculitis, and pediatric inflammatory multisystemic syndrome—that involve immunologic responses following SARS-CoV-2 infection, suggesting the potential existence of underlying immune dysregulations in individuals with COVID-19.

Due to the potential involvement of SARS-CoV-2 infection in cardiopulmonary failure, with its severity being a crucial factor for patients’ overall mortality, extensive evaluation of cardiovascular and respiratory outcomes following COVID-19 infection has been conducted.^2,10^ Although similarities between COVID-19 and several autoimmune diseases have been suggested,^6^ a comprehensive evaluation of autoimmune or inflammatory diseases as postacute COVID-19 sequelae has not yet been established. Thus, this nationwide, population-based study aimed to estimate the incidence and risk of various autoimmune and autoinflammatory connective tissue disorders following COVID-19.

## Methods

### Data Source

This cohort study followed the Strengthening the Reporting of Observational Studies in Epidemiology (STROBE) reporting guideline and was approved by the Korean National Institute for Bioethics Policy with a waiver of informed consent due to the use of deidentified data. We used nationwide, population-based data from the Korea Disease Control and Prevention Agency (KDCA) COVID-19 National Health Insurance Service (NHIS) registry. The NHIS COVID-19 registry is managed by the Korean government and collates information regarding the date of diagnosis, route of infection, and mortality outcomes of individuals with confirmed COVID-19. Korea has a single health care insurance system (NHIS) that covers more than 99% of the entire Korean population and provides comprehensive information regarding socioeconomic status, inpatient and outpatient care, diagnoses of disease, procedures, and prescriptions of the enrolled patients.^11^

### Data Setting and Study Population

**Figure 1.**
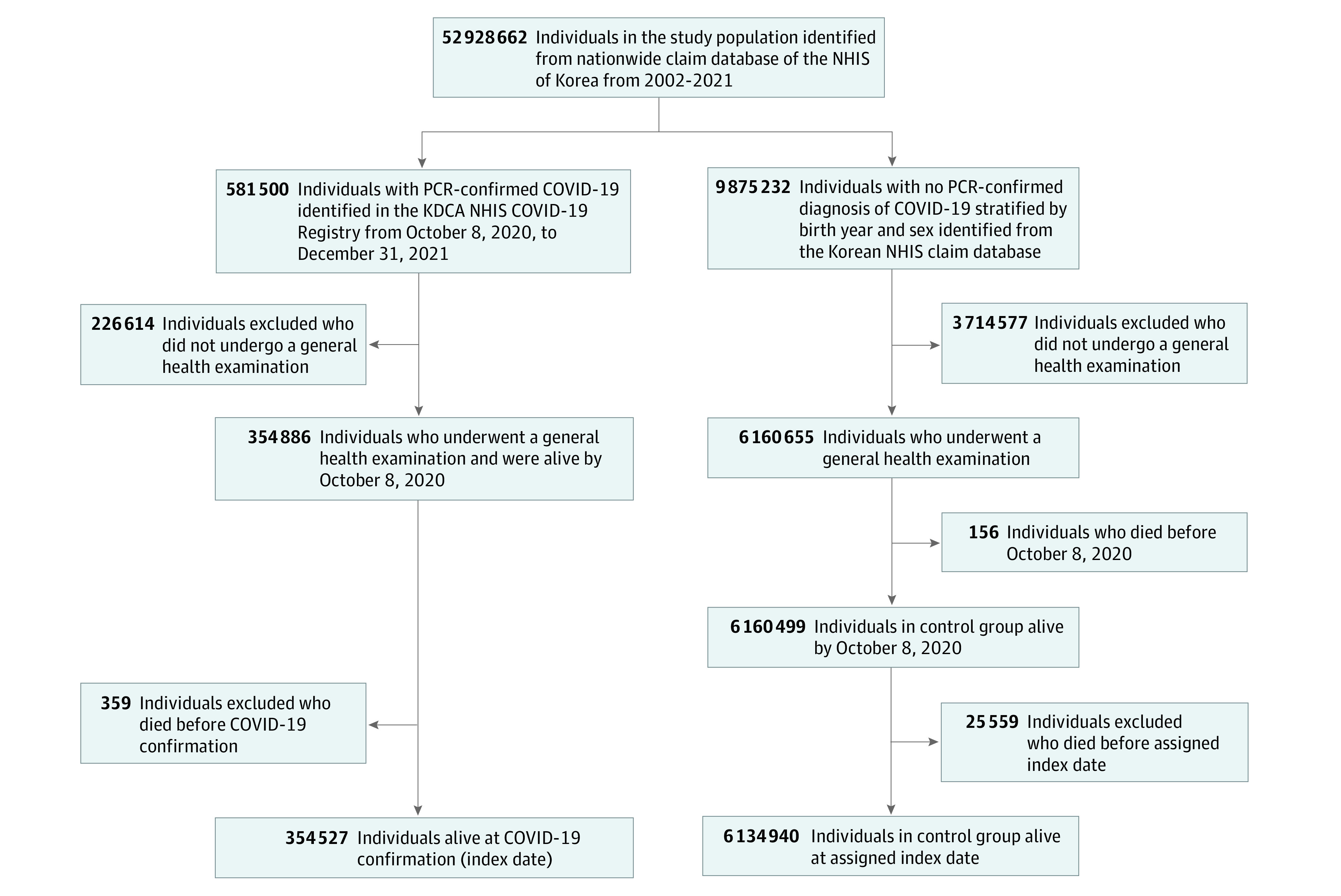
Flowchart of Study Population Selection A total of 354 527 individuals with COVID-19 and 6 134 940 individuals without COVID-19 (as controls) were selected from the Korean Disease Control and Prevention Agency (KDCA) COVID-19 National Health Insurance Service (NHIS) cohort. PCR indicates polymerase chain reaction.

Because Korea was 1 of the last countries to exhibit the nationwide spread of COVID-19, the number of confirmed COVID-19 cases before October 2020 was very small (24 352 cases [0.047%] estimated on the basis of the total population of Korea in 2020).^12,13^ Owing to the data anonymization policy established by the Korean government, our database excluded the information collected on or before October 7, 2020. Among the 581 500 individuals who received a confirmed COVID-19 diagnosis between October 8, 2020, and December 31, 2021, we extracted the data of only those who underwent a general health examination for further covariate control (354 886 individuals). The general health examination is provided by the Korean government annually or biannually to all employees, householders, and citizens aged 40 years or older.^14,15^ It includes health assessments such as blood and urine tests, anthropometric measurements, and also gathers information about an individual’s lifestyle and behavior through structured questionnaires.^15^ Finally, individuals who had tested positive for COVID-19 via polymerase chain reaction testing and were alive at the date of diagnosis were identified (Figure 1). All individuals underwent polymerase chain reaction testing at government-operated triage rooms located across various cities, including both individuals who exhibited symptoms and individuals who were asymptomatic and working or residing in high-risk environments. The date of positive COVID-19 test result served as the study index date for the COVID-19 cohort.

For comparison, we identified 9 875 232 individuals stratified by birth year and sex who had no evidence of SARS-CoV-2 infection (ie, those in the NHIS database who were not registered in the NHIS COVID-19 registry) as the primary control cohort (approximately 20% of the total Korean population) from the entire Korean population in 2020.^13^ Then, we extracted data from only those who had general health examination data (6 160 655 individuals), and those who were alive by October 8, 2020 (6 160 499 individuals). To ensure that the control group had a similar observational period as the COVID-19 group, we randomly assigned the study index date for the control participants according to the distribution of the study index date in the COVID-19 group; hence, the proportion of people enrolled on a certain date was the same in both the control and COVID-19 groups. The study population was followed up from the study index date to each disease diagnosis, emigration, death, or the end of the study period (December 31, 2021).

### Outcomes

The incidences and risks of autoimmune and autoinflammatory connective tissue disorders were assessed during the follow-up of those without a history of such outcomes before the study index date. The occurrence of outcome diseases was defined as at least 3 medical visits with the corresponding *International Statistical Classification of Diseases and Related Health Problems, Tenth Revision (ICD-10) *diagnostic code. To validate our cohort and analyses, outcomes of cardiovascular diseases that were reported to be associated with COVID-19^10^ and outcomes less likely to be associated with COVID-19 were set as the positive and negative control outcomes, respectively, and were examined. The predefined outcomes and corresponding *ICD-10* codes are summarized in eTable 1 in Supplement 1.

### Covariates

The demographics, socioeconomic statuses, lifestyle factors, and comorbidity profiles of the study population were obtained from the NHIS database. We set covariates that may potentially be associated with the disease outcome on the basis of previous literature and the biological plausibility of associations.^10,14,16,17,18^ The predefined covariates are listed in the eMethods in Supplement 1.

### Statistical Analyses

The propensity scores for individuals were estimated as the probability of belonging to the COVID-19 cohort on the basis of the covariates and were used to calculate the inverse probability weights, which were calculated as follows: probability of belonging to the COVID-19 cohort / (1 − the probability of being in the COVID-19 cohort). Covariate balances before and after the application of probability weights were assessed using standardized mean differences. We then estimated the risks of predefined outcomes for COVID-19 vs control cohorts. Statistical estimates were derived using the multivariable Cox proportional hazard analysis after adjusting for all covariates used for inverse probability weighting. For each analysis, individuals who had already received a diagnosis with the target outcome at the index date or before were excluded; hence, the analysis included only individuals at risk. To further investigate specific populations within both groups, we then conducted subgroup analyses according to age, sex, severity of COVID-19 (intensive care unit [ICU] care vs non-ICU care), and COVID-19 vaccination status. Viral vector vaccines (ChAdOx1, Oxford-AstraZeneca; Ad26.COV2.S, Janssen-Johnson & Johnson), mRNA vaccines (BNT162b2, Pfizer-BioNTech; mRNA-1273, Moderna), and protein subunit vaccines (NVX-CoV2373, Novavax) were supplied on a national basis. Vaccination completion was assessed according to the schedules recommended for each vaccine. All statistical analyses were performed using SAS statistical software version 9.4 (SAS Institute) and R statistical software version 3.4.1 (R Project for Statistical Computing) from September 2022 to August 2023.

In sensitivity analyses, given the exclusion of a substantial number of individuals without health examination data, we compared demographic and clinical characteristics between the examined and unexamined populations to assess potential selection bias. Next, we repeated our analysis including only those alive at least 60 days after their COVID-19 diagnosis or index date, with a more stringent observation period considering the lag time in the outcome disease developments. This 60-day threshold was determined by our preliminary analysis, which indicated a markedly elevated mortality rate in the COVID-19 cohort during that period (eFigure 1 in Supplement 1), thereby excluding early mortality cases as a competing risk for outcome disease development.

## Results

### Study Population

In total, 354 527 individuals in the COVID-19 group (mean [SD] age, 52.24 [15.55] years; 179 041 women [50.50%]) and 6 134 940 individuals (mean [SD] age, 52.05 [15.63] years; 3 074 573 women [50.12%]) in the control group were analyzed (Table and Figure 1). Assessment of covariate balance after the application of inverse probability weighting suggested that the covariates were well balanced (eFigure 2 in Supplement 1). The mean (SD) follow-up times for the COVID-19 and control cohorts were 119.70 (117.90) and 121.40 (118.70) days, respectively.

**Table.** Demographic and Clinical Characteristics of Individuals With COVID-19 and Controls Before and After Inverse Probability Treatment Weighting

Characteristic	Preweighting, patients, No. (%)			Postweighting, weighted %		
	COVID-19 (n = 354 527)	Control (n = 6 134 940)	ASD	COVID-19	Control	ASD
Age, mean (SD), y	52.24 (15.55)	52.05 (15.63)	0.0097	52.00 (65.90)	52.00 (16.01)	0.0010
Sex						
Female	179 041 (50.50)	3 074 573 (50.12)	0.0055	50.26	50.25	0.0003
Male	175 486 (49.50)	3 060 367 (49.88)		49.74	49.75	
Insurance type						
Standard	341 665 (96.37)	5 925 309 (96.58)	0.0084	97.11	97.08	0.0015
Medicaid	12 862 (3.63)	209 631 (3.42)		2.89	2.92	
Income level quartile^a^						
1 (Highest)	102 008 (28.77)	1 836 937 (29.94)	0.0342	30.03	30.25	0.0006
2 (Higher)	100 392 (28.32)	1 764 919 (28.77)		29.09	29.06	
3 (Lower)	93 035 (26.24)	1 541 440 (25.13)		25.94	25.39	
4 (Lowest)	57 718 (16.28)	957 348 (15.60)		14.94	15.29	
Area of residence						
Urban	193 943 (54.70)	2 735 002 (44.58)	0.2028	54.39	54.76	0.0074
Rural	160 584 (45.30)	3 399 938 (55.42)		45.61	45.24	
No. of health care encounters per year, mean (SD)	15.90 (16.73)	15.03 (16.75)	0.0514	15.09 (64.96)	14.94 (17.09)	0.0091
COVID-19 vaccination status						
Vaccinated at least 1 time	194 194 (54.78)	3 954 348 (64.46)	0.0221	63.39	63.90	0.0083
Unvaccinated	160 333 (45.22)	2 180 592 (35.54)		36.61	36.10	
General health examination data, mean (SD)						
Height, cm	164.40 (9.36)	164.20 (9.35)	0.0158	164.30 (39.63)	164.30 (9.60)	0.0002
Weight, kg	66.28 (13.37)	65.62 (13.30)	0.0506	65.65 (55.72)	65.66 (13.69)	0.0007
Body mass index^b^	24.40 (3.70)	24.20 (3.92)	0.0540	24.21 (15.41)	24.21 (4.12)	0.0001
Waist circumference, cm	81.95 (11.20)	81.38 (11.77)	0.0504	81.41 (44.73)	81.38 (11.95)	0.0024
Smoking status						
Current smoker	68 162 (19.23)	1 161 460 (18.93)	0.0080	19.04	18.96	0.0020
Former smoker	52 678 (14.86)	1 086 653 (17.71)		17.55	17.54	
Never smoker	233 687 (65.92)	3 886 827 (63.36)		63.41	63.50	
Blood pressure, mean (SD), mm Hg						
Systolic	123.00 (15.14)	123.00 (15.04)	0.0009	123.00 (64.61)	123.00 (15.48)	0.0013
Diastolic	75.88 (10.36)	75.90 (10.20)	0.0015	75.88 (44.10)	75.90 (10.49)	0.0020
Laboratory values, mean (SD)						
Hemoglobin, g/dL	14.17 (1.58)	14.20 (1.59)	0.0177	14.19 (6.72)	14.19 (1.64)	0.0025
Fasting serum glucose, mg/dL	101.60 (25.88)	101.30 (25.14)	0.0122	101.30 (109.50)	101.30 (25.90)	0.0006
Aspartate aminotransferase, U/L	26.68 (25.47)	26.49 (23.70)	0.0075	26.72 (131.20)	26.53 (28.09)	0.0078
Alanine transaminase, U/L	26.30 (28.92)	26.17 (27.39)	0.0049	26.46 (183.40)	26.17 (28.12)	0.0104
γ-Glutamyltransferase, U/L	36.81 (50.82)	36.53 (50.97)	0.0060	36.55 (212.10)	36.54 (52.56)	0.0002
Creatinine, mg/dL	0.86 (0.63)	0.86 (0.53)	0.0097	0.86 (2.70)	0.86 (0.54)	0.0013
Underlying disease						
Hypertension	106 702 (30.1)	1 759 640 (28.68)	0.0290	28.71	28.62	0.0018
Diabetes	61 160 (17.25)	966 435 (15.75)	0.0388	15.78	15.73	0.0013
Dyslipidemia	143 511 (40.48)	2 363 824 (38.53)	0.0374	38.67	38.57	0.0020
Chronic obstructive pulmonary disease	5676 (1.60)	94 600 (1.54)	0.0037	1.50	1.50	0.0002
Chronic kidney disease	4881 (1.38)	68 218 (1.11)	0.0225	1.13	1.11	0.0019
Liver disease	10 113 (2.85)	169 071 (2.76)	0.0056	2.73	2.75	0.0010
Atopic dermatitis	4114 (1.16)	68 358 (1.11)	0.0039	1.12	1.12	0.0004
Allergic rhinitis	17 869 (5.04)	297 662 (4.85)	0.0085	4.87	4.87	0.0001
Asthma	8427 (2.38)	136 555 (2.23)	0.0096	2.23	2.22	0.0009
Hepatitis B	7217 (2.04)	123 402 (2.01)	0.0014	2.02	2.02	0.0003
Hepatitis C	1318 (0.37)	18 817 (0.31)	0.0107	0.31	0.31	0.0002
HIV infection	18 (0.01)	276 (<0.01)	0.0005	0	0	0.0005

Abbreviation: ASD, absolute standardized difference.

SI conversion factors: To convert γ-glutamyltransferase to microkatals per liter, multiply by 0.0167; alanine transaminase to microkatals per liter, multiply by 0.0167; aspartate aminotransferase to microkatals per liter, multiply by 0.0167; creatinine to micromoles per liter, multiply by 76.25; fasting glucose serum, multiply by 0.0555; hemoglobin to grams per liter, multiply by 10.

^a^ The income level was divided into quartiles on the basis of health insurance premiums.

^b^ Body mass index was calculated as weight in kilograms divided by height in meters squared.

### Autoinflammatory and Autoimmune Connective Disorders Following COVID-19

**Figure 2.**
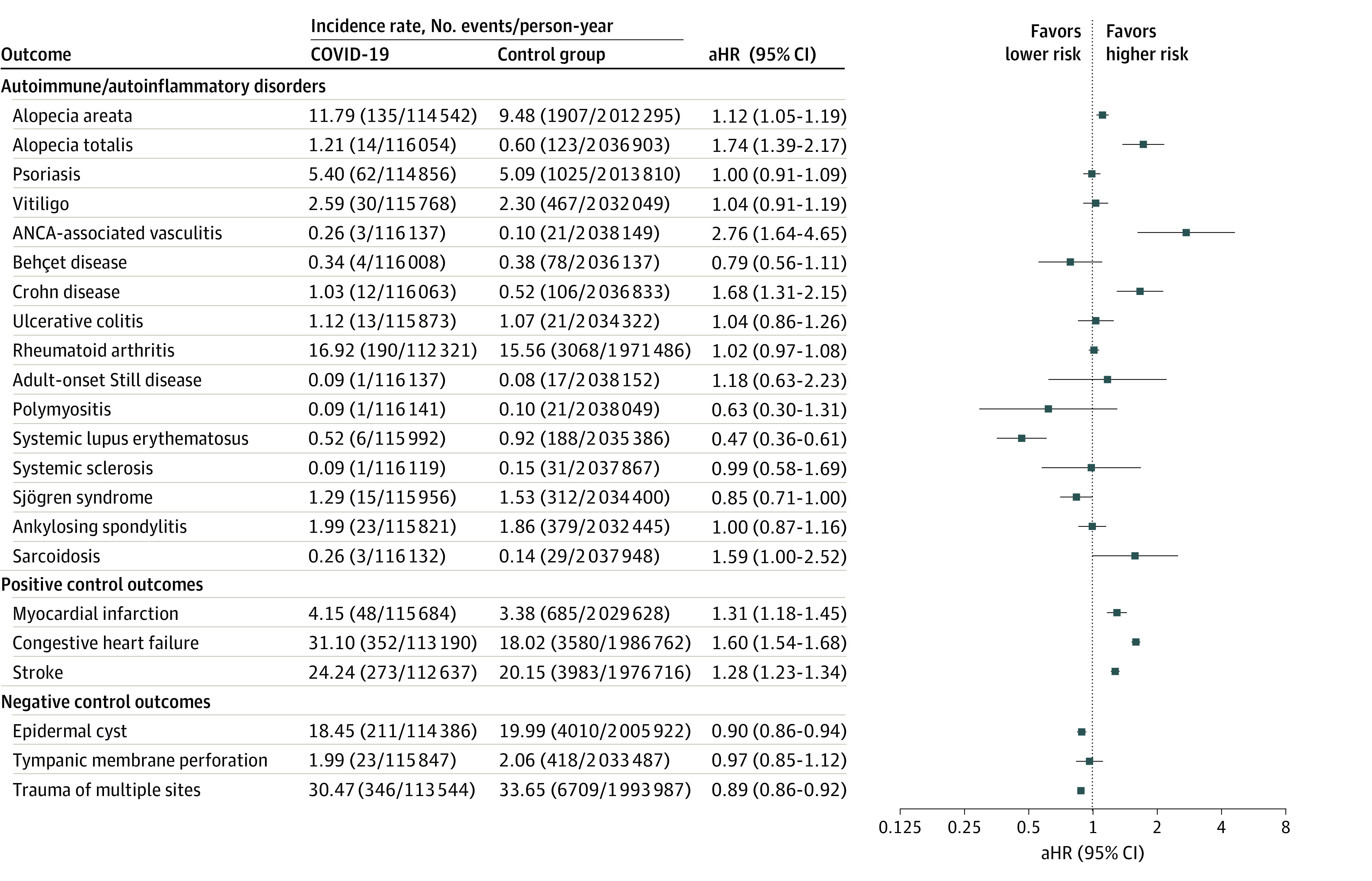
Risks of Incident Autoimmune and Autoinflammatory Disease Outcomes in the COVID-19 Cohort Compared With the Control Cohort The forest plot depicts adjusted hazard ratios (aHRs) and 95% CIs of individuals with COVID-19 compared with control participants. The hazard estimates were adjusted for all 32 covariates used for the inverse probability of treatment weighting. ANCA indicates antineutrophilic cytoplasmic antibody.

The incidences and risks of predefined diseases in the COVID-19 and control cohorts during the follow-up were estimated (Figure 2). Individuals with COVID-19 had significantly higher risks of alopecia areata (adjusted hazard ratio [aHR], 1.12; 95% CI, 1.05-1.19), alopecia totalis (aHR, 1.74; 95% CI, 1.39-2.17), antineutrophil cytoplasmic antibody (ANCA)–associated vasculitis (aHR, 2.76; 95% CI, 1.64-4.65), Crohn disease (aHR, 1.68; 95% CI, 1.31-2.15), and sarcoidosis (aHR, 1.59; 95% CI, 1.00-2.52). However, the risk of SLE was lower (aHR, 0.47; 95% CI, 0.36-0.61) in the COVID-19 cohort. The positive and negative outcome suggested minimal overdetection bias in the COVID-19 cohort. The cumulative incidence of each outcome disease is shown in eFigure 3 in Supplement 1.

### Subgroup Analyses

**Figure 3.**
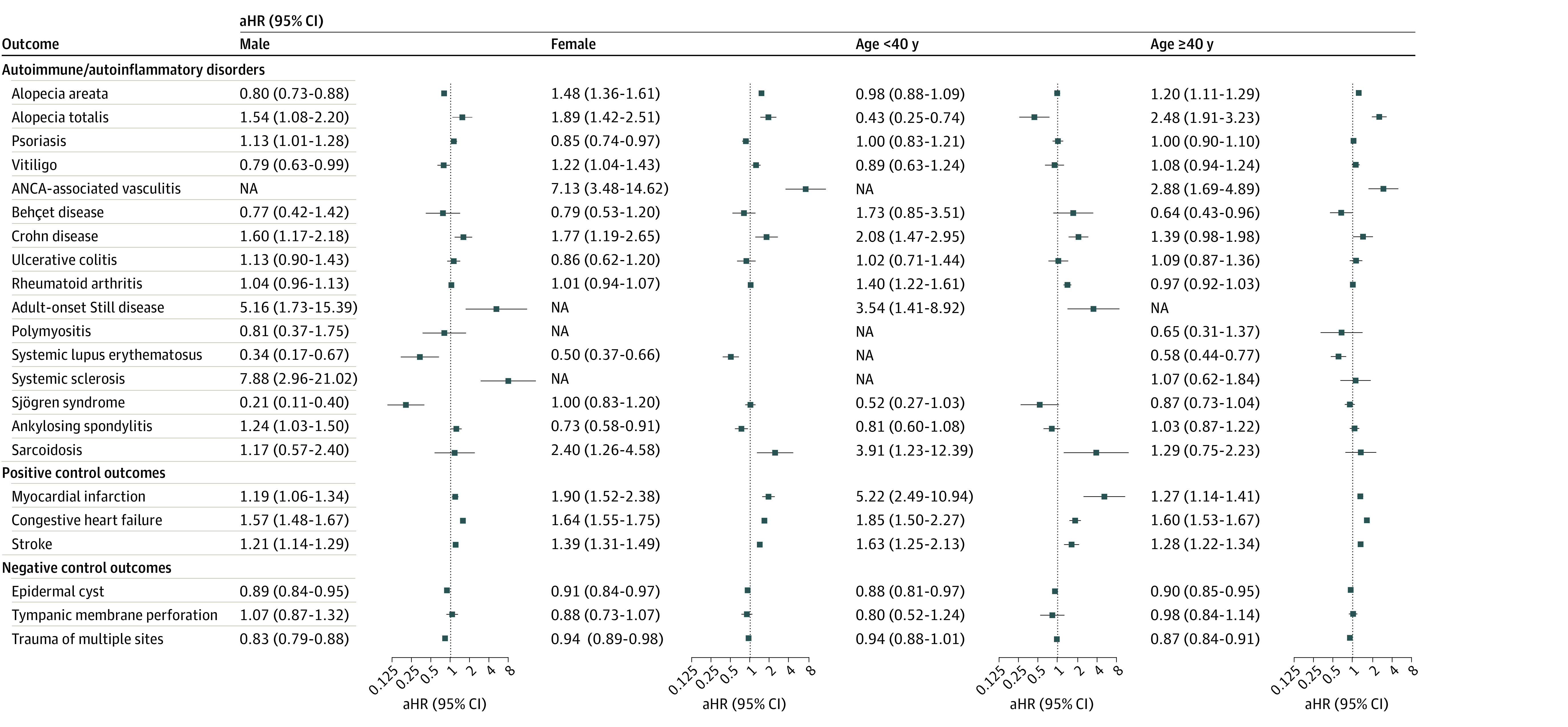
Subgroup Analyses of the Risks of Incident Autoimmune and Autoinflammatory Disease Outcomes Stratified by Age and Sex The forest plot depicts adjusted hazard ratios (aHRs) and 95% CIs of individuals with COVID-19 compared with control participants. The hazard estimates were adjusted for all 32 covariates used for the inverse probability of treatment weighting. ANCA indicates antineutrophilic cytoplasmic antibody; and NA, not available.

**Figure 4.**
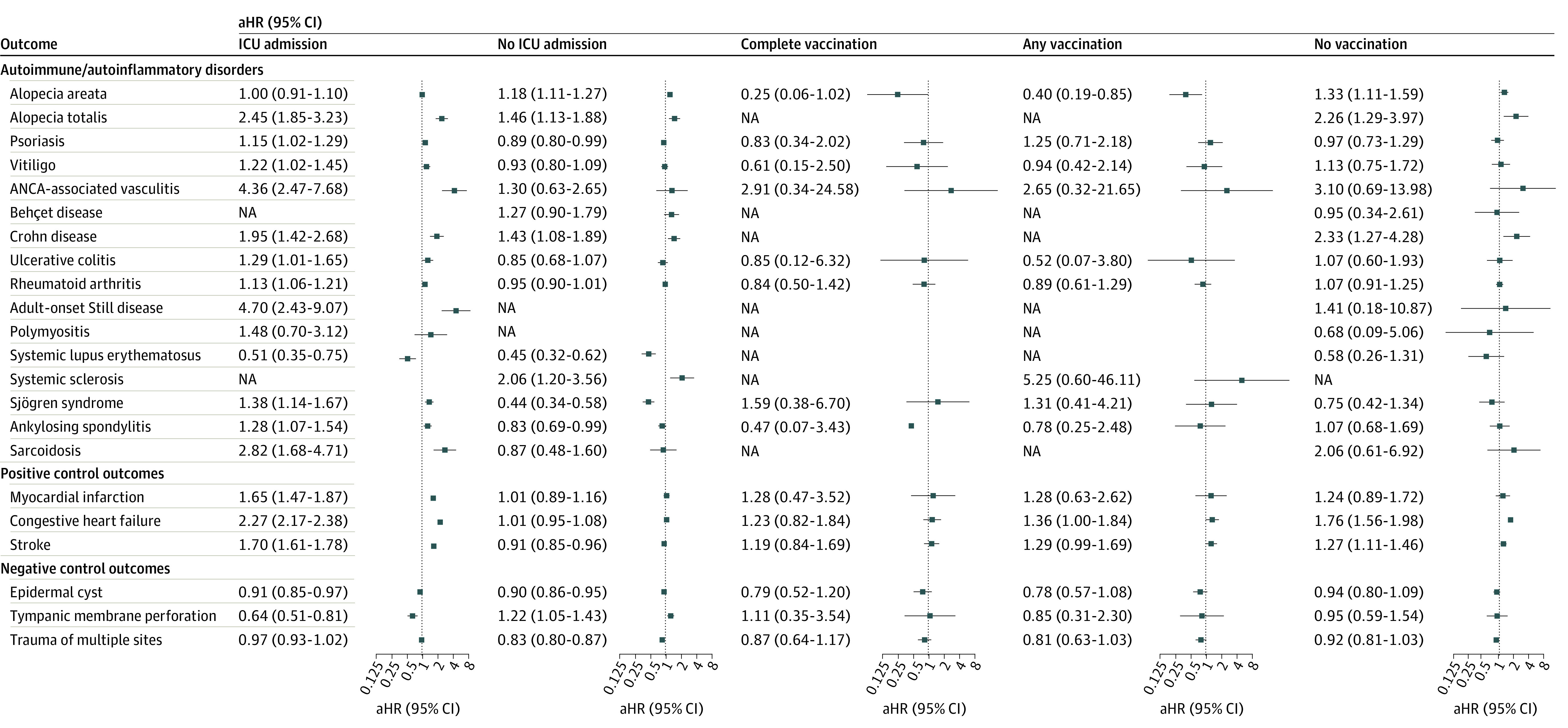
Subgroup Analysis of the Risks of Incident Autoimmune and Autoinflammatory Disease Outcomes in the COVID-19 Cohort Stratified by COVID-19 Severity and COVID-19 Vaccination Status The forest plot depicts adjusted hazard ratios (aHRs) and 95% CIs of individuals with COVID-19 compared with control participants. Subgroup analyses stratified by severity of COVID-19 (intensive care unit [ICU] vs non-ICU) and vaccination status are shown. Vaccination completion was assessed according to the schedules recommended for each vaccine. The hazard estimates were adjusted for 32 covariates used for the inverse probability of treatment weighting. ANCA indicates antineutrophilic cytoplasmic antibody; and NA, not available.

We further examined the risks of incident disease outcomes in the subgroups according to age, sex, and severity of COVID-19 (Figure 3 and Figure 4). The subgroup comprising women showed increased risks of alopecia areata, alopecia totalis, vitiligo, ANCA-associated vasculitis, Crohn disease, and sarcoidosis in the COVID-19 cohort; the subgroup comprising men in the COVID-19 cohort revealed increased risks of alopecia totalis, psoriasis, Crohn disease, adult-onset Still disease, systemic sclerosis, and ankylosing spondylitis. With age stratification, the risks of alopecia areata, totalis, and ANCA-associated vasculitis were higher in individuals aged 40 years or older, whereas the risks of Crohn disease, rheumatoid arthritis, adult-onset Still disease, and sarcoidosis were higher in individuals aged younger than 40 years (Figure 3).

Incident disease outcomes were then evaluated on the basis of the severity of COVID-19 (Figure 4). Despite lower SLE risk in both subgroups and reduced risk of Sjögren syndrome and ankylosing spondylitis in the non-ICU group, the overall risks of incident disease outcomes increased markedly with the severity of the acute stage of COVID-19. When stratified by COVID-19 vaccination status (Figure 4), a greater risk of autoimmune diseases such as alopecia areata, alopecia totalis, and Crohn disease was observed in the unvaccinated group, as well as for positive control outcomes. However, in the vaccinated subgroups, increased risks were diminished for both autoimmune and positive control outcomes.

### Sensitivity Analysis

Demographic data and clinical characteristics were compared between those who underwent health examinations and those who did not (eTable 2 in Supplement 1). The examined group predominantly consisted of adults, because health examinations are generally targeted at householders or employed individuals. The COVID-19 positivity rates were similar for both groups. Notably, the COVID-19 vaccination rate was higher in the examined group. This disparity may be attributed to the public health policy in Korea at that time, which involved a cautious approach to vaccinating adolescents and children due to concerns over efficacy and potential risks. This suggested that our findings were predominantly derived from the adult population.

Sensitivity analyses of disease risks in survivors for at least 60 days after COVID-19 diagnosis were performed (eTable 3 and eFigure 4 in Supplement 1). Furthermore, due to the highest standardized mean difference in area of residence, we conducted an additional analysis adjusting only for this variable (eFigure 5 in Supplement 1). Both sets of findings were consistent with the main results.

## Discussion

In this cohort study, we comprehensively examined and compared the risks of autoimmune and autoinflammatory connective tissue disorders in COVID-19 and control cohorts. Several previous reports have evaluated the incidence of autoimmune diseases following COVID-19.^19,20^ One large cohort study^19^ using a global COVID-19 data set found that the COVID-19 group had a higher risk of various autoimmune diseases compared with a control group. Subgroup analyses stratified by race indicated that White patients generally exhibited a higher risk than Asian patients, except for SLE.^1^^9^ Another study^20^ assessing the incidence of autoimmune diseases after COVID-19 in the German population also observed an elevated risk within the COVID-19 cohort (first onset of autoimmune disease incidence rate ratio, 1.43; 95% CI, 1.37-1.48). Given that our study primarily included Asian participants, we hypothesize that their estimated risks were lower, or may appear to be lower, as a result of delayed disease development or progression, compared with those observed in other ethnic groups. Furthermore, the prevalence of SLE in Korea is lower than that in the US (18.8-21.7 cases per 100 000 people vs 78.5-124.0 cases per 100 000 people)^21^ and the allele frequency of autoimmune disease–associated single-nucleotide variation is more varied among White individuals than in Asian individuals.^22^

In a biological context, one study^23^ has suggested that SARS-CoV-2 infection may be associated with autoimmunity. Widely distributed tissue antigens may be a target of cross-reactive antibodies generated against SARS-CoV-2 epitopes.^23^ Individuals with COVID-19 were reported to have low levels of complements^24^ and positive autoantibodies.^25,26,27,28,29^ Another hypothesis suggested that the release of cytokines (ie, a cytokine storm) may trigger autoimmune responses as bystander activation.^30,31^ Various cytokines, including interleukin (IL)–6 and IL–1, were reported to be associated with immune-mediated damage in individuals with SARS-CoV-2 infection,^32,33^ and the efficacy of antagonists of such cytokines in severely ill patients have been extensively studied.^34,35,36,37^ Dysfunctional angiotensin-converting enzyme 2 and its variant were also suggested to contribute to a skewed inflammatory microenvironment.^38,39^

Patients with COVID-19 mount an early and robust defense through the activation of type 1 and 2 interferon (IFN) responses, which are pivotal against viral infections.^40,41^ Nevertheless, studies^40,41^ indicate that these IFN responses may also induce hyperinflammation, exacerbate the severity of COVID-19, and be associated with mortality. A high IFN status has also been associated with the pathogenesis of autoimmune disorders, such as vitiligo and alopecia areata, which exhibit type 1 and type 2 IFN signatures, respectively,^42,43^ suggesting a potential association of excessive antiviral responses with the subsequent breaching of immune-privileged areas leading to immune responses against self-antigens.

The risk of psoriasis was slightly elevated in the COVID-19 subcohort comprising men and those who had severe COVID-19. Previous reports described aggravation of preexisting psoriatic lesions^44,45^ and increased levels of T helper (T_H_) 17–related cytokines in patients with COVID-19.^46,47^ Thereby, the T_H_17–skewed milieu induced by SARS-CoV-2 infection may contribute to the pathogenesis of psoriasis.

The incidence of ANCA-associated vasculitis was increased in individuals with COVID-19, which was consistent with previous reports.^48,49,50^ Plausible mechanisms involve viral-induced hypercoagulability resulting from endothelial infection or injury, complement system activation, and dysregulation of the coagulation cascade, ultimately leading to vasculopathy.^51,52^ Moreover, the protracted exposure of neutrophil extracellular traps has been postulated to elicit the development of antineutrophil antibodies.^50^

A few studies have reported Crohn disease^53,54^ and ulcerative colitis following COVID-19.^55,56^ Notably, our overall COVID-19 cohort showed increased risk of Crohn disease, whereas the risk of ulcerative colitis was increased in the severe COVID-19 subgroup only. Crohn disease is mainly mediated by T_H_1 and T_H_17, whereas ulcerative colitis is an atypical T_H_2 disease.^57^ Hyperactivated T_H_1 response against SARS-CoV-2 may be associated with Crohn disease, and triggering of T_H_2 response which is associated with poor prognosis of COVID-19 and may contribute to the development of ulcerative colitis.^58^

The precise association of COVID-19 with rheumatoid arthritis remains unclear, and inconsistent findings have been reported in the literature.^59,60,61,62^ Although several investigations^61,62^ failed to detect significant differences in the prevalence of anticitrullinated antibody positivity following COVID-19, some investigations^62^ suggested that cytokine dysregulation is more likely to be associated with rheumatoid arthritis than antibody-mediated reactions. Our data showed that younger individuals (<40 years) and those who had a severe COVID-19 infection had an increased risk of rheumatoid arthritis.

Development of sarcoidosis following COVID-19 infection has also been reported.^63,64^ Although the causes of sarcoidosis are not fully understood, evidence suggests that an aberrant T_H_1 response together with cytokines such as IL–2, IL–12, IL–17, IL–22, IFN–γ, and tumor necrosis factor–α may contribute to sarcoidal granuloma formation.^65,66^ However, we believe that our findings regarding this should be interpreted with caution because we had only a small number of incident cases in the COVID-19 group.

Our data revealed that the risk of incident SLE was decreased in the COVID-19 cohort, which seems to contradict the results of other autoimmune diseases in our study. The observation period may not have been long enough to capture the full spectrum of SLE development. This is suggested by the cumulative incidence of SLE (eFigure 3 in Supplement 1), which shows a delayed disease onset occurring nearly 100 days after a COVID-19 diagnosis. Moreover, SLE may be associated with the severity of COVID-19; a higher mortality in critical COVID-19 cases may skew toward reduced SLE risk.

The severity of the acute stage of COVID-19 was associated with autoimmune disease outcomes. Overall, the risks of the most common outcome diseases were higher among the ICU-admitted subgroup compared with those with milder (non-ICU) cases. The severity of COVID-19 and death were associated with increased levels of diverse cytokines, such as tumor necrosis factor–α and IL-6,^67^ suggesting a possible association with sustained autoimmunity.

Whether COVID-19 vaccination triggers autoimmune diseases has been controversial. Our data showed no elevated disease risks in the vaccinated subgroups. Notably, the risk in positive control outcomes also disappeared, suggesting a potential protective effect of vaccination against COVID-19–related disease development.^68^ This is consistent with a previous report^69^ that evaluated the risk of autoimmune diseases after mRNA vaccination in Korean population, wherein no significant increase in the risk of autoimmune diseases was noted.

Our results emphasize the need to focus on managing not only the acute stages of COVID-19 itself but also autoimmune diseases as complications of COVID-19. Although the risks of developing each disease following COVID-19 varied, there was a clear tendency toward increased risk overall, especially in those who experienced a severe case of COVID-19. This suggests the existence of a common pathway, which may involve excessive cytokine storm leading to prolonged autoimmune responses that trigger specific underlying pathophysiology of each disease. Taken together, surveillance for the new development of autoimmune and autoinflammatory diseases for the myriad of COVID-19 survivors globally is suggested.

### Limitations and Strengths 

This study has several limitations. The demographic composition consisted almost entirely of a single ethnicity, and the age distribution was largely skewed toward adults. Consequently, the results are not generalizable to adolescents and children. Despite rigorous adjustments with covariates, possible misclassifications and residual confounders may exist. There is a possibility that there were some people who had SARS-CoV-2 infection but did not undergo the COVID-19 polymerase chain reaction test and were allocated to the control group. Some diseases included only a small number of incident cases, which could have led to imprecise interpretation. Our data lacked detailed information regarding each participant (eg, information on genetic background) and could not differentiate between individuals who were more susceptible to autoimmune diseases. Early mortality may have acted as a competing factor that could obscure the development of diseases. In addition, the follow-up period was relatively short to fully assess disease development. However, the key strengths of this study lie in its comprehensive statistical analyses, which incorporated diverse covariates with positive and negative controls.

## Conclusions

Our study comprehensively investigated the risks of autoimmune and autoinflammatory connective tissue disorders in patients with COVID-19 compared with controls, highlighting these disorders as potential post–COVID-19 sequelae. Long-term management of patients with COVID-19 should include evaluation of subsequent development of autoimmune and autoinflammatory connective tissue disorders.

## Notes

Supplement 1. **eTable 1. ***International Statistical Classification of Diseases, Tenth Revision (ICD-10)* Codes of the Included Diseases **eMethods.** The Predefined Covariates for Inverse Probability Weighting **eFigure 1.** Cumulative Incidence Plot for All-Cause Mortality Among Patients With COVID-19 and Controls **eFigure 2.** Covariate Balance After Application of Inverse Probability Weighting **eFigure 3**. Cumulative Incidences of Autoinflammatory and Autoimmune Connective Tissue Disorders Outcomes **eTable 2.** Sensitivity Analysis: Demographic and Clinical Characteristics of Individuals Who Underwent and Who Did Not Undergo General Health Examination **eTable 3.** Sensitivity Analysis: Demographic and Clinical Characteristics of Individuals Who Survived at Least 60 Days Post COVID-19 Diagnosis and Controls Before And After Weighting **eFigure 4.** Sensitivity Analyses: Risks of Incident Autoimmune and Autoinflammatory Disease Outcomes in Individuals Survived by at Least 60 Days Post–COVID-19 Diagnosis and the Controls **eFigure 5.** Sensitivity Analysis: Risks of Incident Autoimmune and Autoinflammatory Disease Outcomes in COVID-19 Cohort and the Control Cohort, Adjusted for Area of Residency (Urban vs Rural) Status Only

Supplement 2.
Data Sharing Statement
