# Supplementary material for: Autoimmune and Autoinflammatory Connective Tissue Disorders Following COVID-19
Source: JAMA Netw Open. 2023 Oct 6;6(10):e2336120. doi: 10.1001/jamanetworkopen.2023.36120 (PMC10559181; doi:10.1001/jamanetworkopen.2023.36120)
Supplement: Supplement 1. — eTable 1. International Statistical Classification of Diseases, Tenth Revision (ICD-10) Codes of the Included Diseases eMethods. The Predefined Covariates for Inverse Probability Weighting eFigure 1. Cumulative Incidence Plot for All-Cause Mortality Among Patients With COVID-19 and Controls eFigure 2. Covariate Balance After Application of Inverse Probability Weighting eFigure 3. Cumulative Incidences of Autoinflammatory and Autoimmune Connective Tissue Disorders Outcomes eTable 2. Sensitivity Analysis: Demographic and Clinical Characteristics of Individuals Who Underwent and Who Did Not Undergo General Health Examination eTable 3. Sensitivity Analysis: Demographic and Clinical Characteristics of Individuals Who Survived at Least 60 Days Post COVID-19 Diagnosis and Controls Before And After Weighting eFigure 4. Sensitivity Analyses: Risks of Incident Autoimmune and Autoinflammatory Disease Outcomes in Individuals Survived by at Least 60 Days Post–COVID-19 Diagnosis and the Controls eFigure 5. Sensitivity Analysis: Risks of Incident Autoimmune and Autoinflammatory Disease Outcomes in COVID-19 Cohort and the Control Cohort, Adjusted for Area of Residency (Urban vs Rural) Status Only [file jamanetwopen-e2336120-s001.pdf]

Supplemental Online Content

Lim SH, Ju HJ, Han JH, et al. Autoimmune and autoinflammatory connective tissue disorders following COVID-19. *JAMA Netw Open*. 2023;6(10):e2336120. doi:10.1001/jamanetworkopen.2023.36120

eTable 1. *International Statistical Classification of Diseases, Tenth Revision* (*ICD-10*) Codes of the Included Diseases

eMethods. The Predefined Covariates for Inverse Probability Weighting

eFigure 1. Cumulative Incidence Plot for All-Cause Mortality Among Patients With Covid-19 and Controls

eFigure 2. Covariate Balance After Application of Inverse Probability Weighting

eFigure 3. Cumulative Incidences of Autoinflammatory and Autoimmune Connective Tissue Disorders Outcomes

eTable 2. Sensitivity Analysis: Demographic and Clinical Characteristics of Individuals Who Underwent and Who Did Not Undergo General Health Examination

eTable 3. Sensitivity Analysis: Demographic and Clinical Characteristics of Individuals Who Survived at Least 60 Days Post COVID-19 Diagnosis and Controls Before And After Weighting

eFigure 4. Sensitivity Analyses: Risks Of Incident Autoimmune and Autoinflammatory Disease Outcomes in Individuals Survived by 60 Days Post–COVID-19 Diagnosis and the Controls

eFigure 5. Sensitivity Analysis: Risks of Incident Autoimmune and Autoinflammatory Disease Outcomes in COVID-19 Cohort and the Control Cohort, Adjusted for Area of Residency (Urban vs Rural) Status Only

This supplemental material has been provided by the authors to give readers additional information about their work.

© 2023 Lim SH et al. *JAMA Network Open*

eTable 1. ***International Statistical Classification of Diseases, tenth revision*** **(ICD-10) codes of the included diseases**

Disease	ICD-10 code
**Autoimmune and autoinflammatory outcomes**	
Alopecia areata	L63
Alopecia totalis	L630, L631
Psoriasis	L40
Vitiligo	L80
Anti-neutrophil cytoplasmic antibody-associated vasculitis	M301, M313, M317, M318
Behcet disease	M352
Crohn disease	K50
Ulcerative colitis	K51
Rheumatoid arthritis	M05, M06, M08
Adult-onset Still disease	M061
Polymyositis	M332
Systemic lupus erythematosus	M32
Systemic sclerosis	M34
Sjogren syndrome	M350
Ankylosing spondylitis	M45
Sarcoidosis	D86
**Positive control outcomes**	
Myocardial infarction	I21
Congestive heart failure	I50
Stroke	I60, I61, I62, I63
**Negative control outcomes**	
Epidermal cyst	L720
Tympanic membrane perforation	H72
Trauma of multiple site	T00
**Chronic diseases that served as the covariates**	
Hypertension	I10
Diabetes mellitus	E08, E09, E10, E11, E13
Dyslipidemia	E78
Chronic obstructive pulmonary disease	J44
Chronic kidney disease	N18
Mild liver disease	K73, K18
Atopic dermatitis	L20
Allergic rhinitis	J30
Asthma	J45
Hepatitis B virus infection	B180, B181, B191
Hepatitis C virus infection	B182, B192
Human immunodeficiency virus infection	B20

© 2023 Lim SH et al. *JAMA Network Open*

eMethods. The predefined covariates for inverse probability weighting

Age at the study index date, sex, income level, insurance type, area of residence, height, weight, body mass index, waist circumference, smoking status, average number of healthcare encounters per year, systolic blood pressure, diastolic blood pressure, serum levels of hemoglobin, fasting blood glucose, liver enzyme levels (aspartate aminotransferase, alanine transaminase, gamma-glutamyl transferase), and creatinine, COVID-19 vaccination status (more than one time vs. none) at index date, and personal histories of hypertension, diabetes mellitus, dyslipidemia, chronic obstructive pulmonary disease, chronic kidney disease, mild liver disease, atopic dermatitis, allergic rhinitis, asthma, hepatitis B and C infection, and human immunodeficiency virus infection.

© 2023 Lim SH et al. *JAMA Network Open*

eFigure 1. Cumulative incidence plot for all-cause mortality among patients with COVID-19 and controls

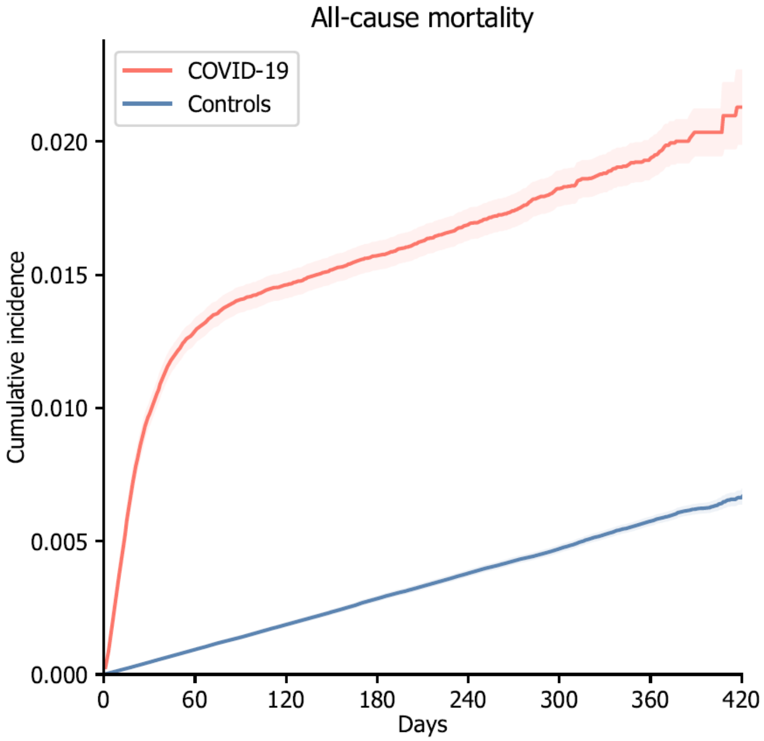

© 2023 Lim SH et al. *JAMA Network Open*

eFigure 2. Covariate balance after application of inverse probability weighting

A

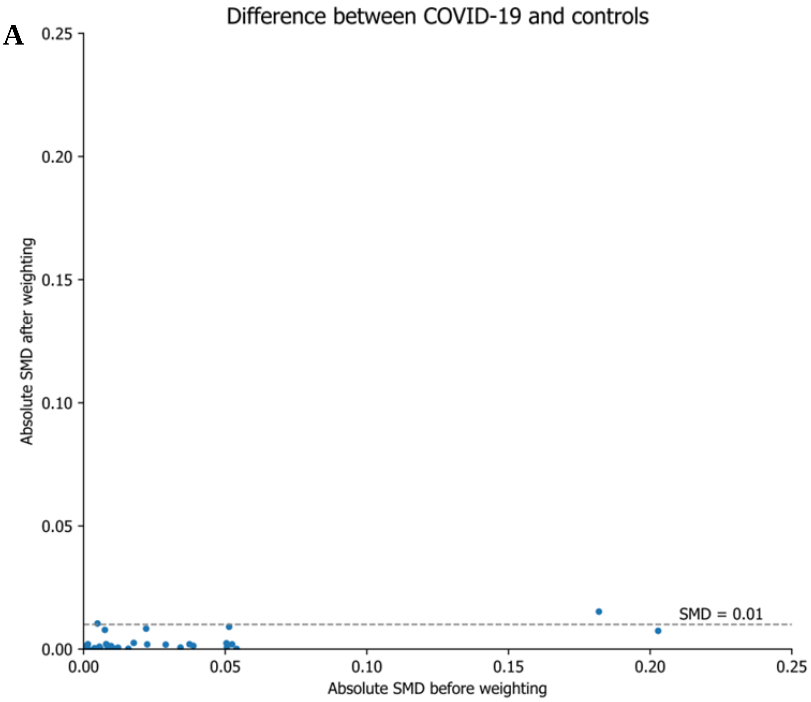

SMDs between individuals with COVID-19 and controls. Abbreviations: COVID-19, Coronavirus 2019; SMD, standardized mean difference.

© 2023 Lim SH et al. *JAMA Network Open*

eFigure 3. Cumulative incidences of autoinflammatory and autoimmune connective tissue disorders outcomes

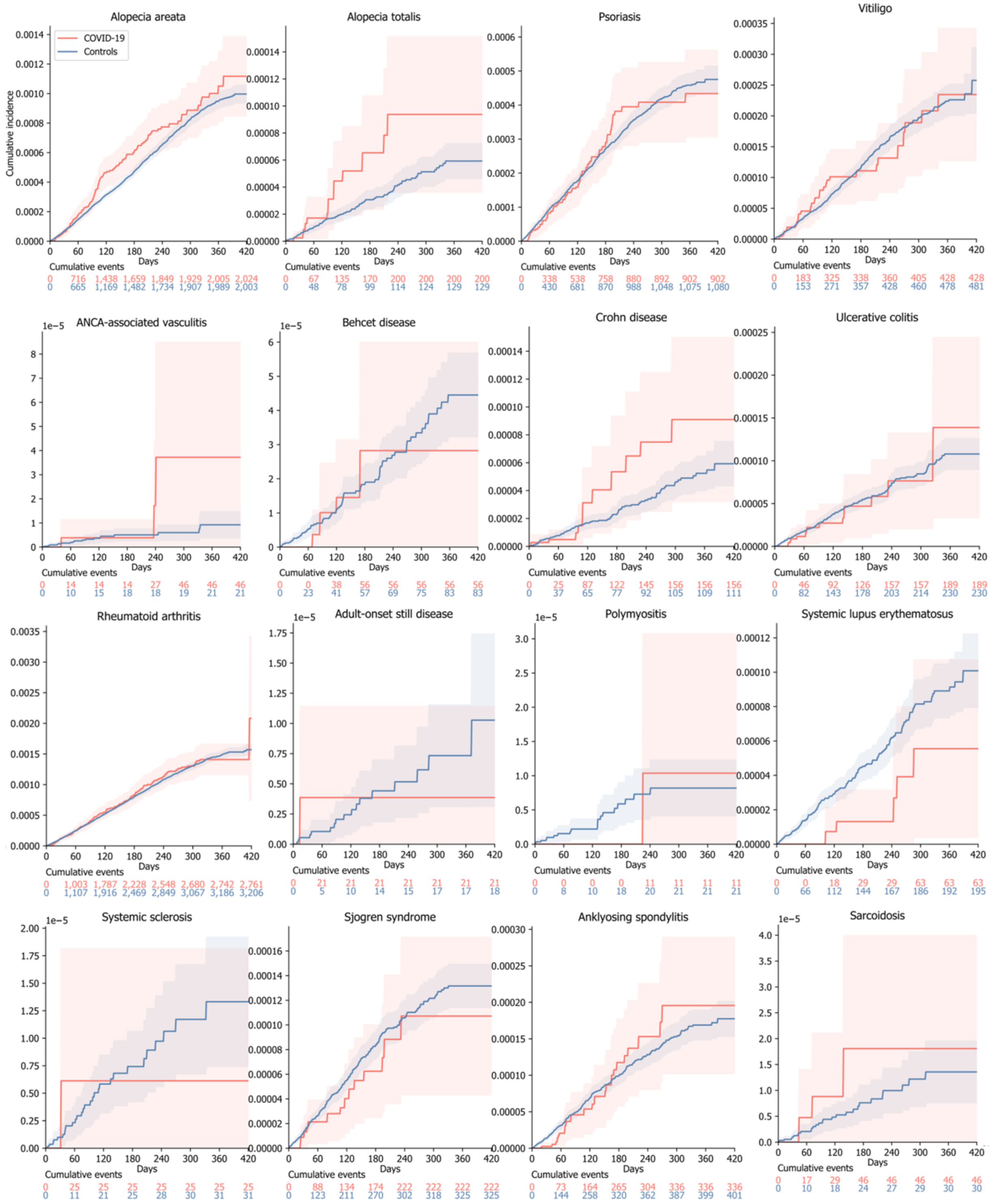

ANCA, anti-neutrophil cytoplasmic antibody

© 2023 Lim SH et al. *JAMA Network Open*

eTable 2. Sensitivity analysis: Demographic and clinical characteristics of individuals who underwent and who did not undergo general health examination

	Individuals who had GE (N=6,482,630)	Individuals who did not undergo GE (N=3,935,099)
**Age, mean ± SD, y**	52.04±15.61	24.76±19.02
≥40	4,875,146(75.2)	735,269(18.68)
18–39	1,607,460(24.8)	1,477,043(37.54)
<18	24(0.00)	1,722,787(43.78)
**Female sex, n (%)**	3,250,599(50.14)	1,794,011(45.59)
**Insurance type, n (%)**		
**Standard**	6,260,919(96.58)	3,744,567(95.16)
**Medicaid**	221,711(3.42)	190,532(4.84)
**Income level, n (%)**		
**Highest**	1,936,598(29.87)	1,333,489(33.89)
**Higher**	1,863,693(28.75)	1,109,109(28.19)
**Lower**	1,633,174(25.19)	832,628(21.16)
**Lowest**	1,013,520(15.63)	622,140(15.81)
**No. of healthcare encounters per year, mean ± SD**	15.06±16.73	11.08±11.76
**COVID-19 positivity**	354,527(5.47)	225,430(5.73)
**COVID-19 vaccination status, n (%)**		
**Vaccinated at least 1 time**	4,253,039(65.61)	1,304,263(33.14)
**Non-vaccinated**	2,229,591(34.39)	2,630,836(66.86)
**Underlying disease, n (%)**		
**Hypertension**	1,862,600(28.73)	252,363(6.41)
**Diabetes mellitus**	1,025,359(15.82)	137,762(3.50)
**Dyslipidemia**	2,504,551(38.63)	312,785(7.95)
**Chronic obstructive pulmonary disease**	99,476(1.53)	16,546(0.42)
**Chronic kidney disease**	72,701(1.12)	16,687(0.42)
**Liver disease**	179,135(2.76)	22,366(0.57)
**Atopic dermatitis**	72,465(1.12)	152,449(3.87)
**Allergic rhinitis**	315,292(4.86)	289,582(7.36)
**Asthma**	144,695(2.23)	134,343(3.41)
**Hepatitis B**	130,414(2.01)	14,075(0.36)
**Hepatitis C**	20,083(0.31)	2,957(0.08)
**HIV infection**	292(0.00)	148(0.00)

Abbreviations: GE, general health examination; HIV, human immunodeficiency virus

© 2023 Lim SH et al. *JAMA Network Open*

eTable 3. Sensitivity analysis: Demographic and clinical characteristics of individuals who survived at least 60 days post COVID-19 diagnosis and controls before and after weighting

	Pre-weighting			Post-weighting		
	COVID-19 (N= 192,854)	Control (N= 3,372,056)	Absolute standardized difference	COVID-19 (%)	Control (%)	Absolute standardized difference
**Age, mean ± SD, y**	49.89 ± 15.00	52.06 ± 15.62	0.1423	51.63 ± 65.58	51.85 ± 15.98	0.0143
**Female sex, n (%)**	95,023 (49.27)	1,692,429 (50.19)	0.0188	(49.87)	(50.16)	0.0059
**Insurance type, n (%)**			0.0039			0.0000
**Standard**	186,574 (96.74)	3,256,561 (96.57)		(97.26)	(97.08)	
**Medicaid**	6,280 (3.26)	115,495 (3.43)		(2.74)	(2.92)	
**Income level, n (%)**			0.0471			0.0080
**Highest**	53,512 (27.75)	1,009,728 (29.94)		(28.52)	(30.24)	
**Higher**	55,418 (28.74)	969,975 (28.77)		(29.58)	(29.06)	
**Lower**	52,930 (27.45)	848,159 (25.15)		(27.32)	(25.42)	
**Lowest**	30,300 (15.71)	525,457 (15.58)		(14.58)	(15.27)	
**Area of residence, n (%)**			0.1587			0.0116
**Urban**	101,303 (52.53)	1,503,271 (44.58)		(43.24)	(45.3)	
**Rural**	91,551 (47.47)	1,868,785 (55.42)		(56.76)	(54.70)	
**COVID-19 vaccination status, n (%)**			0.0547			0.0039
**Vaccinated at least 1 time**	57,598 (29.87)	1,385,341 (41.08)		(37.30)	(40.48)	
**Non-vaccinated**	135,256 (70.13)	1,986,715 (58.92)		(62.70)	(59.52)	
**General health examination data, mean ± SD**						
**Height, cm**	165.00 ± 9.32	164.20 ± 9.36	0.0865	164.40 ± 39.99	164.30 ± 9.60	0.0081
**Weight, cm**	66.93 ± 13.69	65.61 ± 13.30	0.0981	65.81 ± 56.32	65.68 ± 13.71	0.0092
**Body mass index, kg/m^2**	24.44 ± 3.76	24.20 ± 4.09	0.0612	24.23 ± 15.47	24.21 ± 4.26	0.0061
**Waist circumference, cm**	81.81 ± 11.13	81.37 ± 11.66	0.0396	81.44 ± 45.14	81.37 ± 11.84	0.0062
**Smoking, n (%)**			0.0130			0.0038
**Current smoker**	37,544 (19.47)	638,947 (18.95)		(19.24)	(18.98)	
**Ex-smoker**	30,759 (15.95)	596,142 (17.68)		(18.95)	(17.49)	
**Never smoker**	124,551 (64.58)	2,136,967 (63.37)		(61.81)	(63.53)	
**Systolic blood pressure, mmHg**	122.40 ± 14.95	123.00 ± 15.04	0.0362	122.90 ± 64.80	122.90 ± 15.47	0.0003
**Diastolic blood pressure, mmHg**	75.88 ± 10.38	75.90 ± 10.20	0.0018	75.90 ± 44.30	75.90 ± 10.49	0.0002
**Hemoglobin, g/dL**	14.24 ± 1.59	14.20 ± 1.59	0.0248	14.20 ± 6.77	14.20 ± 1.64	0.0035
**Fasting serum glucose, mmol/L**	100.90 ± 25.46	101.30 ± 25.22	0.0148	101.20 ± 109.90	101.30 ± 25.93	0.0028
**Aspartate aminotransferase, U/L**	26.59 ± 25.99	26.49 ± 24.07	0.0041	26.64 ± 102.00	26.50 ± 25.98	0.0055
**Alanine transaminase, U/L**	26.66 ± 28.42	26.16 ± 27.90	0.0177	26.50 ± 157.50	26.18 ± 28.71	0.0114
**Gamma-glutamyl transferase, U/L**	37.29 ± 50.67	36.51 ± 50.94	0.0159	36.68 ± 209.5	36.55 ± 52.58	0.0026
**Creatinine, mg/dL**	0.86 ± 0.62	0.86 ± 0.53	0.0045	0.86 ± 2.24	0.86 ± 0.55	0.0004
**Underlying disease, n (%)**						
**Hypertension**	50,341 (26.1)	959,304 (28.45)	0.0520	(24.77)	(28.38)	0.0043
**Diabetes mellitus**	28,911 (14.99)	527,236 (15.64)	0.0176	(13.64)	(15.61)	0.0037
**Dyslipidemia**	70,442 (36.53)	1,291,408 (38.3)	0.0363	(34.66)	(38.33)	0.0052
**Chronic obstructive pulmonary disease**	2,325 (1.21)	51,209 (1.52)	0.0271	(1.13)	(1.48)	0.0002
**Chronic kidney disease**	1,888 (0.98)	36,593 (1.09)	0.0110	(0.82)	(1.08)	0.0026
**Liver disease**	5,061 (2.62)	91,496 (2.71)	0.0050	(2.49)	(2.71)	0.0032
**Atopic dermatitis**	2,136 (1.11)	37,101 (1.1)	0.0004	(1.07)	(1.10)	0.0010
**Allergic rhinitis**	9,531 (4.94)	163,240 (4.84)	0.0054	(4.76)	(4.86)	0.0007
**Asthma**	4,123 (2.14)	74,762 (2.22)	0.0051	(2.00)	(2.21)	0.0013
**Hepatitis B**	3,717 (1.93)	67,555 (2)	0.0052	(1.90)	(2.01)	0.0009
**Hepatitis C**	651 (0.34)	10,349 (0.31)	0.0058	(0.28)	(0.31)	0.0017
**HIV infection**	13 (0.01)	155 (0.0)	0.0024	(0.01)	(0.00)	0.0006

Abbreviations, COVID-19, Coronavirus 2019; HIV, human immunodeficiency virus

© 2023 Lim SH et al. *JAMA Network Open*

eFigure 4. Sensitivity analyses: risks of incident autoimmune and autoinflammatory disease outcomes in individuals survived by 60 days post COVID-19 diagnosis and the control cohort

	Incidence rate (no. of events/person-years)			
	**COVID-19**	**Controls**	aHR	(95% CI)
**Autoimmune/inflammatory disorders**				
Alopecia areata	12.86 (134/104,205)	10.07 (1,846/1,833,977)	1.16	(1.09-1.24)
Alopecia totalis	1.33 (14/105,604)	0.64 (119/1,856,377)	1.82	(1.46-2.28)
Psoriasis	5.84 (61/104,537)	5.35 (982/1,835,338)	1.06	(0.97-1.16)
Vitiligo	2.66 (28/105,347)	2.41 (446/1,851,960)	1.01	(0.88-1.15)
ANCA-associated vasculitis	0.28 (3/105,683)	0.11 (20/1,857,509)	3.17	(1.87-5.40)
Behcet disease	0.38 (4/105,563)	0.42 (78/1,855,675)	0.80	(0.57-1.13)
Crohn disease	1.14 (12/105,614)	0.54 (101/1,856,313)	1.78	(1.38-2.28)
Ulcerative colitis	1.23 (13/105,439)	1.15 (213/1,854,041)	1.10	(0.91-1.33)
Rheumatoid arthritis	17.79 (182/102,288)	16.51 (2,967/1,796,876)	1.03	(0.98-1.09)
Adult-onset still disease	0.09 (1/105,681)	0.09 (16/1,857,508)	1.28	(0.67-2.44)
Polymyositis	0.09 (1/105,685)	0.11 (21/1,857,417)	0.64	(0.30-1.34)
Systemic lupus erythematosus	0.57 (6/105,552)	0.99 (184/1,854,995)	0.49	(0.38-0.64)
Systemic sclerosis	0.09 (1/105,665)	0.17 (31/1,857,251)	1.03	(0.60-1.74)
Sjogren syndrome	1.42 (15/105,521)	1.66 (307/1,854,102)	0.90	(0.76-1.07)
Anklyosing spondylitis	2.18 (23/105,398)	1.97 (364/1,852,324)	1.06	(0.92-1.23)
Sarcoidosis	0.28 (3/105,678)	0.15 (28/1,857,324)	1.66	(1.04-2.64)
**Positive control outcomes**				
Myocardial infarction	4.46 (47/105,282)	3.65 (676/1,849,749)	1.35	(1.22-1.50)
Congestive heart failure	32.39 (334/103,113)	19.28 (3,492/1,810,767)	1.65	(1.57-1.72)
Stroke	24.93 (256/102,681)	21.30 (3,837/1,801,651)	1.32	(1.26-1.38)
**Negative control outcomes**				
Epidermal cyst	19.40 (202/104,098)	20.39 (3,728/1,828,213)	0.93	(0.89-0.98)
Tympanic membrane perforation	1.99 (21/105,418)	2.10 (390/1,853,271)	1.01	(0.87-1.17)
Trauma of multiple sites	31.55 (326/103,342)	34.30 (6,233/1,817,458)	0.92	(0.88-0.95)

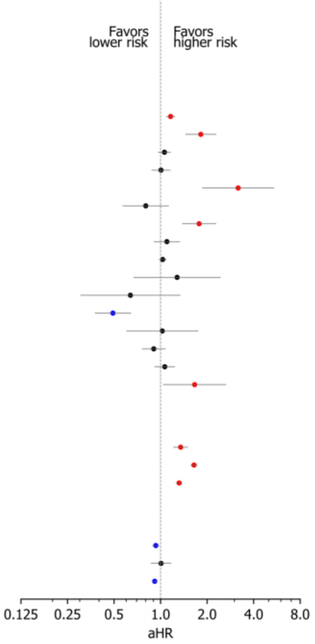

The forest plot depicts aHRs and 95% CIs in individuals with COVID-19 compared with controls. The hazard estimates were adjusted for all 32 covariates used for the inverse probability of treatment weighting. For each analysis, individuals who had already been diagnosed with the target outcome at the index date or before were excluded.

Abbreviations: COVID-19, coronavirus disease 2019; aHR, adjusted hazard ratio; CI, confidence interval.

© 2023 Lim SH et al. *JAMA Network Open*

eFigure 5. Sensitivity analysis: risks of incident autoimmune and autoinflammatory disease outcomes in COVID-19 cohort and the control cohort, adjusted for area of residency (urban vs rural) status only

	Incidence rate (no. of events/person-years)			
	**COVID-19**	**Controls**	aHR	(95% CI)
**Autoimmune/inflammatory disorders**				
Alopecia areata	11.79 (135/114,542)	9.48 (1,907/2,012,295)	1.18	(1.11-1.25)
Alopecia totalis	1.21 (14/116,054)	0.60 (123/2,036,903)	1.82	(1.46-2.27)
Psoriasis	5.40 (62/114,856)	5.09 (1,025/2,013,810)	0.98	(0.90-1.07)
Vitiligo	2.59 (30/115,768)	2.30 (467/2,032,049)	1.02	(0.90-1.16)
ANCA-associated vasculitis	0.26 (3/116,137)	0.10 (21/2,038,149)	2.59	(1.55-4.34)
Behcet disease	0.34 (4/116,008)	0.38 (78/2,036,137)	0.82	(0.58-1.14)
Crohn disease	1.03 (12/116,063)	0.52 (106/2,036,833)	1.68	(1.31-2.14)
Ulcerative colitis	1.12 (13/115,873)	1.07 (218/2,034,322)	1.03	(0.85-1.25)
Rheumatoid arthritis	16.92 (190/112,321)	15.56 (3,068/1,971,486)	1.00	(0.95-1.05)
Adult-onset still disease	0.09 (1/116,137)	0.08 (17/2,038,152)	1.26	(0.67-2.36)
Polymyositis	0.09 (1/116,141)	0.10 (21/2,038,049)	0.59	(0.28-1.24)
Systemic lupus erythematosus	0.52 (6/115,992)	0.92 (188/2,035,386)	0.47	(0.36-0.61)
Systemic sclerosis	0.09 (1/116,119)	0.15 (31/2,037,867)	0.89	(0.53-1.51)
Sjogren syndrome	1.29 (15/115,956)	1.53 (312/2,034,400)	0.80	(0.67-0.94)
Anklyosing spondylitis	1.99 (23/115,821)	1.86 (379/2,032,445)	0.99	(0.86-1.15)
Sarcoidosis	0.26 (3/116,132)	0.14 (29/2,037,948)	1.69	(1.07-2.67)
**Positive control outcomes**				
Myocardial infarction	4.15 (48/115,684)	3.38 (685/2,029,628)	1.20	(1.08-1.33)
Congestive heart failure	31.10 (352/113,190)	18.02 (3,580/1,986,762)	1.47	(1.41-1.53)
Stroke	24.24 (273/112,637)	20.15 (3,983/1,976,716)	1.15	(1.10-1.20)
**Negative control outcomes**				
Epidermal cyst	18.45 (211/114,386)	19.99 (4,010/2,005,922)	0.90	(0.86-0.94)
Tympanic membrane perforation	1.99 (23/115,847)	2.06 (418/2,033,487)	0.93	(0.81-1.07)
Trauma of multiple sites	30.47 (346/113,544)	33.65 (6,709/1,993,987)	0.88	(0.85-0.91)

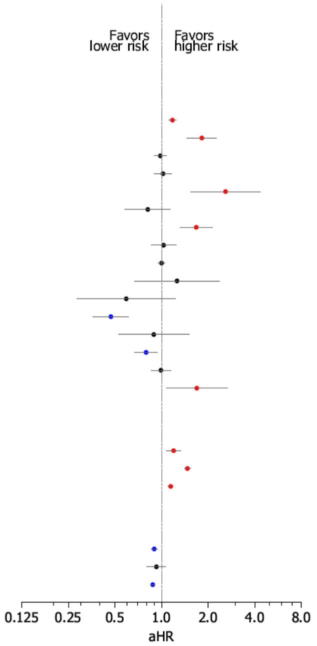

The forest plot depicts aHRs and 95% CIs in individuals with COVID-19 compared with controls. Abbreviations: COVID-19, coronavirus disease 2019; aHR, adjusted hazard ratio; CI, confidence interval.

© 2023 Lim SH et al. *JAMA Network Open*
