# Supplementary material for: Autoimmune and Autoinflammatory Connective Tissue Disorders Following COVID-19
Source: JAMA Netw Open. 2023 Oct 6;6(10):e2336120. doi: 10.1001/jamanetworkopen.2023.36120 (PMC10559181; doi:10.1001/jamanetworkopen.2023.36120)
Supplement: Supplement 2. — Data Sharing Statement [file jamanetwopen-e2336120-s002.pdf]

Data Sharing Statement

Lim. Autoimmune and Autoinflammatory Connective Tissue Disorders Following COVID-19. *JAMA Netw Open*. Published October 06, 2023. doi:10.1001/jamanetworkopen.2023.36120

Data
Data available: No

Additional Information
Explanation for why data not available: The data that support the findings of this study are available from the National Health Insurance Service of Korea, but restrictions apply to the availability of these data, which were used under license for the current study, and so are not publicly available. Data are however available from the authors upon reasonable request and with permission of the National Health Insurance Service of Korea.
